# Advisory teams on the streets: A nurse’s experience report

**DOI:** 10.1590/1980-220X-REEUSP-2022-0026en

**Published:** 2022-07-18

**Authors:** Tatiana Ferraz de Araújo Alecrim, Pedro Fredemir Palha, Jaqueline Garcia de Almeida Ballestero, Simone Teresinha Protti-Zanatta

**Affiliations:** 1 Universidade Federal de São Carlos, Programa de Pós-Graduação em Enfermagem, São Carlos, SP, Brazil.; 2 Universidade de São Paulo, Escola de Enfermagem de Ribeirão Preto, Departamento Materno-Infantil e Saúde Pública, Ribeirão Preto, SP, Brazil.

**Keywords:** Nurse, Nursing Care, Homeless Persons, Primary Health Care, Health Policy, Enfermera, Cuidado de Enfermería, Personas sin Hogar, Asistencia Primaria de Salud, Política de Salud Pública, Enfermeiras e Enfermeiros, Cuidados de Enfermagem, Pessoas em Situação de Rua, Atenção Primária à Saúde, Política de Saúde

## Abstract

**Objective:**

To report a nurse’s work experience with the street medical consultation teams in the city of São Paulo/SP Brazil.

**Method:**

Descriptive, experience report study, which describes the care for homeless people, from a nurse’s perspective and experience.

**Results:**

Among the attributions of the nurses working with the street medical consultation teams, there are the accurate knowledge of the territory, the ability to build bonds, the performance of diagnoses of health and epidemiological conditions, the planning of the team’s actions, the establishment of integration flows with the Health Care Network, the knowledge and understanding about the people ending up on the streets, the supervision of the actions of nursing assistants and community health agents.

**Conclusion:**

Acting as a nurse on the street medical consultation team is a new and challenging experience that requires dynamic, strategic, creative, and empathic actions. The presence of nurses in the Street Medical Consultation teams contributes to ensuring access to health services and comprehensive care, expanding the possibilities of early detection, treatment, monitoring, and healing of chronic and infectious diseases.

## RESUMO

**Objetivo:**

Relatar a experiência acerca da atuação da/o enfermeira/o junto às equipes de consultório na rua no município de São Paulo/SP Brasil.

**Método:**

Estudo descritivo, do tipo relato de experiência, que descreve o cuidado às pessoas em situação de rua, a partir do olhar e da vivência de uma enfermeira.

**Resultados:**

Dentre as atribuições das/os enfermeiras/os que atuavam junto às equipes de consultório na rua, destacavam-se o conhecimento acurado do território, habilidade para construção de vínculos, realização de diagnósticos das condições de saúde e epidemiológico, planejamento das ações da equipe, estabelecimento de fluxos de integração junto à Rede de Assistência à Saúde, conhecimento e compreensão sobre a chegada das pessoas às ruas, supervisão das ações dos auxiliares de enfermagem e agentes comunitários de saúde.

**Conclusão:**

Atuar como enfermeira/o na equipe de consultório na rua é uma experiência nova e desafiadora que requer dos profissionais ações dinâmicas, estratégicas, criativas e empáticas. A presença de enfermeiras/os nas equipes de Consultório na Rua contribui para a garantia do acesso aos serviços de saúde e cuidado integral, amplia as possibilidades de detecção precoce, tratamento, acompanhamento e a cura de agravos crônicos e infectocontagiosos.

## RESUMEN

**Objetivo:**

Relatar la experiencia acerca de la actuación de la/del enfermera/o con los equipos de consultorio en la calle en el municipio de São Paulo/SP, Brasil.

**Método:**

Estudio descriptivo, del tipo relato de experiencia que describe el cuidado a las personas que viven en la calle desde el punto de vista y de la vivencia de una enfermera.

**Resultados:**

Entre las atribuciones de las/de los enfermeras/os que actuaban con los equipos de consultorio en la calle se destacaban el profundo conocimiento del territorio, habilidad para construcción de vínculos, realización de diagnósticos de las condiciones de salud y epidemiológico, planificación de las acciones del equipo, establecimiento de flujos de integración con la Red de Asistencia a la Salud, conocimiento y comprensión sobre la llegada de personas a la calle, supervisión de las acciones de los auxiliares de enfermería y agentes de salud comunitaria.

**Conclusión:**

Actuar como enfermera/o en el equipo de consultorio en la calle es una experiencia nueva y retadora que requiere de los profesionales acciones dinámicas, estratégicas, creativas y empáticas. La presencia de enfermeras/os en los equipos de Consultorio en la calle contribuye para garantizar el acceso a los servicios de salud y cuidado integral, amplía las posibilidades de detección precoz, tratamiento, acompañamiento y la cura de agravios crónicos e infectocontagiosas.

## INTRODUCTION

Social inequities are present in History, revealing that living on the street is a reality that remains over the years. In Brazil, with the abolition of slavery in 1888, the freed ex-slaves and their descendants were the first to use the streets as a space for housing and subsistence. The end of slavery dumped men, women, and children in urban centers who, without jobs, housing, or conditions to provide for their survival, wandered through the cities begging or being subjected to unworthy working conditions^(1,2)^.

Despite having existed for more than a century, the homeless population (HP) is not accounted for by demographic censuses, since data collection is based on households. Although being invisible to the census operation, this population becomes more and more visible on the cities’ streets and squares^(3)^.

It was only in 2008 that HP was counted, through a specific national population census. The survey was carried out in only 71 municipalities in Brazil by the Ministry of Social Development and Fight against Hunger. It identified 31,922 people over 18 years of age living on the streets. Of these, 82% were male; 67% black, aged between 25 and 44 years; 58.6% declared having an occupation, such as collector of recyclable materials (27.5%), car watchers (14.1%), construction workers (6.3%), and loading assistants; despite this, 19% do not have access to even one meal a day. It should be noted that not all Brazilian municipalities and large capitals such as São Paulo, Belo Horizonte and Recife were not included, as they had carried out similar surveys in previous years^(4)^.

In 2015, the estimate of people living on the streets in Brazil was 101,854; however, it is not possible to measure the real number of this population due to the lack of periodicity, methodological procedure, and national guidelines for census applications. In view of this, HP counting occurs according to the initiatives of each municipality^(5,6)^.

In this regard, the census carried out in the city of São Paulo in 2022 showed 31.884 people living on the streets, revealing an increase of 31% of this population compared to the result of the same census carried out in 2019. Current data for the city of São Paulo corroborate the results of the national survey regarding gender identity, age, and job, but they showed an increase in family nuclei and people living in tents. In addition, it is noteworthy that 92.3% of these people wish to stop living on the streets^(7)^.

National Decree No. 7053, of December 23, 2009, defines HP as:

“heterogeneous population group that has in common extreme poverty, interrupted or weakened family ties, and the lack of regular conventional housing, and that uses public places and degraded areas as a space for housing and livelihood, temporarily or permanently, as well as accommodation centers for temporary overnight stays or as temporary housing”^(8)^.

Although there are several reasons influencing people ending up on the streets, the effects of living on the street are similar for all of them. In common, these people are on the margins of society; they are victims of violence and prejudice; they are socially, emotionally, and physically vulnerable; they suffer deprivation and do not have their basic life needs met; thus, they need to develop survival strategies on a daily basis^(5)^.

When compared to the general population, the population of homeless people has a five to ten times greater risk of death and a 48 to 67 times greater risk of having tuberculosis. Problems such as chronic diseases, mental health problems, abusive use of psychoactive substances, foot problems, infestations, sexually transmitted infections, HIV and AIDS, oral health problems and high-risk pregnancy are described as the main health problems of this population^(9,10,11)^.

Access to information and health for HP is scarce and insufficient; settling in a specific geographic space is a challenge, given the need for daily movements in search of food, work, rest and overnight, they are considered a highly vulnerable population. Therefore, one can state that the homeless population has a greater need for health care, requiring services that articulate their practices and guarantee them comprehensive care through public policies and actions that respond to their needs^(11–13)^.

Although the achievements established by public policies for the HP are still incipient, they signal to the importance of the National Policy for the Homeless Population (*PNPSR*), in 2009. This policy contributed to the implementation of the Street Medical Consultation (*CnaR*) in 2011, as a Primary Care team (*eAB*) for a specific population, through the publication of the National Primary Care Policy (*PNAB*)^(14–17)^.

The *CnaR* teams’ main objective is to expand HP’s access to health, given the *in loco* comprehensive care for the HP, continued care on an itinerant basis, addressing their different health needs and, when necessary, the use of Primary Health Units (*UBS*) facilities. Actions are planned during the day and/or night and on any day of the week, having as a parameter a Street Medical Consultation team for every eighty to one thousand people living on the streets^(11,16)^.

The teams can be formed by: nurses; nursing technicians or assistants; psychologists; social workers; occupational therapists; physicians; dental surgeons; technician or assistant in oral health; physical education professional/teacher; professional with training in art and education; social agent and community health agents, the last two being high school-level professionals. According to the number and professional composition, the teams are classified into modalities I, II and III, with modality III being the only one that integrates the medical professional; thus, it is worth noting the need for the territory when choosing the modality to be implemented^(18)^.

In 2004, the Municipal Health Department of São Paulo started the implementation of the Community Agents Program (*PACS*) with the HP, aiming at promoting, preventing, detecting, and treating the most frequent diseases of this population. In 2008, the *PACS* teams were transformed and expanded into Family Health teams. Then, the Special Family Health Teams (FHS S) were created, operating in regions with the highest concentration of HP. Some *UBS*s were adapted and designed to serve these users, with infrastructure such as a place for bathing. In August 2012, the FHS S, in compliance with the new guidelines of the Ministry of Health, were converted to *CnaR* ^(19)^.

Currently, the Municipality of São Paulo has 26 teams of Street Medical Consultation Offices, modality III. Each team is linked to a *UBS*, consisting of one or more nurse(s). Among the specific duties of the nurse(s) established by the *PNAB*, the following are highlighted: care for individuals and families in all life cycles; extramural nursing consultations, such as on streets, in schools or homes; articulation of collective activities; request for complementary exams; medication prescription and referrals according to established protocols and technical regulations; planning and management of activities; permanent education and participation in the management of supplies^(17,19)^.

Given the above, the construction of care with the HP requires the nurse(s) to act with the creation of bonds that humanize each encounter, by understanding without judging, as well as respecting and establishing limits. It is essential to work in teams that permanently re-signify their actions, in which continued care is established in the time of the other, starting from small steps^(11,20)^.

Considering the history, the high health and social vulnerability presented, the need for comprehensive and specialized care, the importance of adding more scientific production with this theme, the insertion of nurse(s) in the management and production of care for people living on the streets, this article aims to report the experience regarding nurses’ work with the Street Medical Consultation Office teams in the city of São Paulo/SP, Brazil.

## METHOD

### Design of Study

This is a professional experience report with qualitative characteristics, of a descriptive nature, with a cross-sectional time frame.

### Local

The present professional experience report describes nurses’ work and the care of people living on streets with the teams of the Street Medical Consultation Office in the central region of the city of São Paulo, especially in the territories known as “old center” and “crackland”. The description is made from the perspective and experience of a nurse who, from 2009 to 2022, has worked with these teams in different settings, such as in the implementation of teams, research, care and service management.

The city of São Paulo is the capital of the state of São Paulo; it stands out as the main financial, corporate, and commercial center in South America. Data from the Brazilian Institute of Geography and Statistics (IBGE) for 2021 estimate a population of 12,396,372 inhabitants in an area of 1,523 km^2^, as the capital of business, cultural, and gastronomic diversity and which concentrates the largest number of people living on the streets in Brazil^(7)^.

The main characteristics of the territories on which the experience is based are the concentration of tourist attractions, large architectural structures, trade routes, bus terminals, train and subway stations, thousands of passers-by during the day, important financial institutions, business centers, government agencies, churches, non-governmental organizations (NGOs), and degraded areas.

The municipality, in its history, presents an increasing number of HP and, consequently, the increase of *CnaR* teams. Nurses play a pivotal role in these teams; thus, this report describes the actions of direct assistance, care coordination, and supervision performed by the nurse who works at *CnaR*.

### Ethical Aspects

The present professional experience report reflects the impressions of the authors of this text about the experience described and discussed in the sections of this article. It contains the analysis of conceptual implications, description of work processes, flows, routines, and intervention strategies in the care of the homeless population in the city of São Paulo. As it did not require any form of data collection, there was no need for submission to the ethics committee.

## RESULTS

When talking about the care practice of the nurse(s) who work with the Street Medical Consultation teams, it is worth starting with the need for accurate knowledge of the territory. Territorialization is a fundamental instrument for organizing work processes and health surveillance. On the street, the territory is dynamic and does not depend on geographic limits. It takes place daily in the places of passage, in the points of fixation, in the different forms of subsistence, in the agreements and disagreements. Thus, it is essential to consider the relationship that the individual establishes with this territorial space, understanding that in the face of difficulties, weaknesses and risks of each territory, it is also essential to know its potentials and its resources.

The mapping carried out by the *CnaR* teams is essential to recognize the disposition of the homeless population in each of the places they occupy. Reality needs to be known *in loco*. The culture of migration is part of being on the street; the lack of fixation in a permanent physical space makes it difficult to establish care. However, depending on the territory, geographic migration is less constant, which allows the location of individuals for the continuity of actions.

Getting closer to homeless people can be considered one of the greatest challenges. To be on the street is to be exposed to all forms of absences, deprivation and violence, it is to be a victim of prejudice and exclusion. Interaction hardly happens in the first approaches, making the construction of bonds a slow process and the result of intense persistence. It is observed that these individuals, for the most part, have stories of intense physical and emotional suffering, family conflicts, abandonment, compulsory removals, and violation of rights.

The process of building bonds, fundamental for the construction of comprehensive and longitudinal care, occurs gradually, based on a lot of insistence, patience, and constant presence of the teams in the places with the highest concentration of homeless people. The interaction and approach are necessary for the registration of the individual in the *CnaR* team. It is common for those registered to disclose the team’s work. Through “good advertising”, “word of mouth”, they contribute to the approximation and bonding of street friends to the team. The role of the nurse on the street requires establishing an interpersonal relationship based on bonding, qualified listening, empathy, welcoming and respect for diversity. Thus, trust and the establishment of a bond are made in daily care actions.

The registration of people living on the streets is not always done at the first meeting; the routine expulsion of HP from the spaces they occupy, social exclusion, and prejudice make them afraid and suspicious of the professionals’ approaches. Thus, having the same team members, if possible in pairs, at the same places and times, makes them a reference to be sought by HP when they feel the need. The registration is individual, made by the health agent; after this register, the medical record is opened, where all care will be recorded; the medical records are filed at the reference Primary Health Unit.

Ensuring that the registered person is accompanied by the reference health agent in the first consultation at the *UBS* is essential. This practice strengthens the bond, promotes equity of access, and contributes to the understanding that the *UBS* is a universal space for care. Considering that the experience of HP’s time is unique, there is a need and emergency to meet their needs in a moment that is today and now; people living on the streets can be judged for not being able to wait and for being short-sighted; however, it is worth noting that the immediacy of this population is closely related to the uncertainty of their lives longevity and the constant need to guarantee their survival.

Being in a health service waiting for care can arouse curious and often disapproving looks towards HP, due to their unconventional physical appearance, worn clothes, and unpleasant odor. The time spent waiting to be served can, for example, result in missing the time for food distribution by social facilities, churches, and NGOs known as “boca de rango” (slang expression meaning place where to find food). Between waiting for care and eating, the basic human need for nutrition is prioritized.

Thus, the guarantee of brief care and the appreciation of their presence in the health service, the provision of all care resources available in a single visit to the *UBS*, such as shared consultation with the multidisciplinary team, vaccination, collection of exams, dressings, baths, dispensing and administration of medications, identification of potential managing counselors, referrals, and delivery of the Brazilian Public Health System (*SUS*) card have been important strategies for comprehensive care and bond strengthening. Although there is routine loss and the need to issue more than one copy, the *SUS* card is a very desired and valued document by the HP, since this identification and health document is made and delivered on time, with no need for proof of address or presentation. of preexisting documents. The card issue using self-declared data and the absence of bureaucracy contributes significantly to the feeling of belonging to the Primary Health Unit.

The night period is a time of great exposure and greater risk for homeless people; violence, the use of psychoactive substances, and low temperatures increase during the night, making it a period of permanent alert and difficulty for sleeping/rest. It is at dawn, when the city becomes busier, that HP generally feels safer and more protected to sleep; thus, many fall asleep more soundly during this period. Faced with this reality, waking them up first thing in the morning is not opportune; one has to be sensitive to recognize the best moment to make the approach.

Diagnosing the homeless population’s health conditions is essential to identify the necessary interventions for comprehensive care. It is necessary to understand health in its expanded concept, that is, the one related to living conditions, work, education, food, housing, safety, sleep/rest, leisure, among others. Thus, in the face of HP’s care, social aspects most often overlap care demands, since these individuals do not have the minimum conditions to meet their basic human needs decently.

In this context, it is essential for the nurse to plan the team’s actions and establish integration flows with the Health Care Network (*RAS*), discuss cases in technical meetings with the service network, carry out street visits and in social facilities, participate in systematic meetings with the service team according to the demands presented, and articulate actions with the territory’s intersectoral network.

Health care for the homeless population involves understanding who the people to be cared for are, how they got to the street, how they survive on it, and what their main health needs are. This way, it is possible to build individual and collective care strategies, whether on the street itself, at the reference *UBS,* or in other facilities. Considering the demands presented, understood as this “package” of desires defined by users and health care professionals, procedures are offered, such as the collection of material for laboratory tests, dressings, measurement of blood pressure and capillary blood glucose, administration of oral and injectable medications, as well as directly observed therapy for tuberculosis^(21)^.

The epidemiological scenario has shown the prevalence of health problems that call the nurse(s) to knowledge and the development of skills to act in a general way in the face of non-communicable chronic diseases, in the active search for pregnant women and promotion of prenatal care, in the regular monitoring of minor children and adolescents, in the active search for screening, monitoring and treatment of tuberculosis, in the provision of counseling and rapid testing of AIDS, syphilis, hepatitis and other infectious diseases, in the monitoring of people with psychological conditions, and people who abuse alcohol, *crack* and other drugs, in carrying out nursing consultations, collective activities such as educational groups, and in active participation in continuing education processes^(11)^.

The nurse is also responsible for supervising the activities and closing the productions of nursing assistants/technicians and health agents, for evaluating and discussing job log data, holding team meetings to plan visits, as well as actions in health services and territory. They are also directly responsible for organizing and running campaigns, forecasting and providing materials and supplies for procedures, organizing individual/collective assistance, completing and building reports, as well as representing the *CnaR* team at meetings.

Thus, they play a fundamental role in the articulation of the health care network and in the intensification of intersectoral actions. Regular meetings for discussions and referrals are essential, such as matrix support with the Psychosocial Assistance Center for Alcohol and Drugs (*CAPS AD*), the Child Psychosocial Assistance Center (*CAPS IJ*), and the Adult Psychosocial Assistance Center (*CAPS Adult*), teams that work in the *UBS*s and Family Health Teams (FHSs), Specialized Reference Center for Social Assistance (*CREAS*), Specialized Reference Center for Homeless People (*CREAS POP*), NGOs, churches, Shelter Center managers, among others. Considering HP’s diverse needs, for care to be truly comprehensive, the strengthening of institutional partnerships is essential.

The diversities and challenges involved in HP’s care go beyond the difficulty of accessing public policies, the prejudice and social stigma of those who are homeless. The professionals who provide this care are also targets of prejudice, as if working with this population is a demerit or lack of option in the job market; therefore, the presence of nurses who, in addition to technical, care and management skills, have the profile to build narratives to overcome these obstacles, is required.

## DISCUSSION

The Street Medical Consultation Office can be described as:

“Absence of walls, inexistence of a table, meeting in moving places, the sun, the wind, the cold, the heat, the rain, often accompanied by dirt and a strong odor. Talking about health in the midst of the drug scene, being in contact with life stories from/in distressing contexts, are events that require a unique exercise marked by the construction and deconstruction of values”^(22)^.

Faced with the current work scenario, the nurse of the Street Medical Consultation Office team is faced with numerous challenges, diversities ranging from guaranteeing access to health services to raising community awareness, in the quest to contribute to the understanding that homeless people are subjects with rights and that they shall be respected. This way, professionals working at *CnaR* need to be attentive to identify the biological, psychological, spiritual, and social needs of their members^(13,22)^.

Although HP occupies the busiest regions of the city of São Paulo, its invisibility is marked by the absence of effective public policies meeting its real needs; it is urgent to develop forms of care and actions that value the experiences of each of these individuals. For this practice, it is essential to build innovative forms of care that go beyond the biomedical model and achieve a practice of empathic listening with an inter and transdisciplinary bias^(12,23)^.

Among the specificities of the work in the *CnaR*, it is important that nurses have profile and are available to create bonds and are prepared to hear the word no, since the fear and insecurity shown by the HP hinder the approach and inclusion for care in the first approaches. The interpersonal relationship contributes positively to mutual respect and trust, embracement, access, and adherence to treatment^(13,20,23)^.

In the search for practices that produce care on the streets, the *CnaR’s* nurses act creatively based on the knowledge and deep recognition of the geographic territory and its epidemiological needs. Territorialization is essential to know the street and its spaces, the overpasses, squares, holes, *malocas* (communal dwellings), marquees, meeting points, places of permanence, for shelter and for work. It is common for groups to approach to each other and organize themselves by similarities; for example, alcohol users generally do not group with crack users, family groups approach each other, teenagers join other teenagers, recyclable collectors approach others who perform the same activity^(8,11,12)^.

Besides the individual care such as consultations, clinical assessments, referrals, and procedures, collective activities such as groups and campaigns are resources that are widely used by *CnaR* teams. These activities have as their main objective guidance and health promotion; they are carried out on the street itself, viaducts, sidewalks, squares or in accomodation centers, better known as “albergues”^(8,11,22)^.

Given the high risk of HP having tuberculosis, actions aimed at the active search for respiratory symptoms, diagnosis and treatment of TB are carried out in a systematic way: guidance groups, smear collection, and directly observed medication administration can be carried out in the street itself, depending on the needs of each person registered and the characteristics of the territory. It is worth noting that, when the situation requires it, CnaR teams also use the practice of assisted medication for treatments other than TB, such as supervised administration of antibiotic therapy, psychotropics, and continuous use medications^(24)^.

Aiming at promoting health, some teams invest in cultural and leisure activities. The itinerant toy library takes place in street spaces, where donated toys in good conditions are used by children and adults to address health issues in a playful way. When some skill is identified among the participants, they are encouraged to produce and share their knowledge with others; an example is the practice of juggling. The cinematheque takes place in NGO spaces; movies chosen from suggestions and selections made by the health team and the HP are shown; during the movie presentation, popcorn and juices collected from the workers themselves are offered. In the drawing workshops, sheets of paper, crayons and colored pencils are available for free and/or thematic drawings; there are also visits to tourist attractions such as libraries, parks and museums, places in front of which homeless people often sleep, but do not know their internal spaces^(11,14,20)^.

Working with the homeless population is to understand that the guarantee of rights to/in life also permeates the experience of death in life and life in death. Dealing with the death of those who in life had their rights violated and who, in the face of death, will not even have the right to have their names described on the graves is also a theme present in the daily lives of the teams. These invisibles in life and indigents at the time of departure are registered in the teams with their first and last name, age, occupation, cities of origin, place of settlement and overnight stay, time living on the street, where they eat, and health information; the nurse(s), teams and street colleagues know their stories, their memories, their pain, their dreams and their joys^(8,12,14)^.

That being said, the deaths of homeless people are immediately reported to the nurse. Knowing that a registered person can be buried as a pauper moves the entire team in the effort to ensure that their companions are not buried as unknown people; thus, medical records, *SUS* card, and test results are gathered to be presented to the legal medical institute (IML) and, if necessary, even body recognition is performed. Actions to guarantee rights such as these strengthen the bond of teams in the territory, increase access and adherence to health care^(15)^.

The systematization and production of the data presented here is a limitation of this study, as they are based on the narrative and reflections of the professional practice exclusively experienced by a nurse. In this regard, it can be inferred that the data are not watertight, as they can generate new perceptions and interpretations when narrated and reflected by other professionals and even other nurses. Therefore, the results obtained here shall not be generalized.

## CONCLUSION

The singularities and adversities that make up HP care permeate issues that go beyond the health sector; it also faces the lack of access to public policies, misery, prejudice, exclusion, and social stigma. Thus, the presence of the nurse(s) is essential, as they are the ones who, besides having technical, care, and managerial skills, are available to build narratives that overcome these obstacles.

For that reason, we have to highlight the importance of nurses and health professionals who are dynamic, strategic, creative and empathetic in the construction of practices and knowledge promoting integral and longitudinal health care, to be carried out in the most diverse urban spaces. It should also be noted that the role of nurses in the Street Medical Consultation Office teams in the central region of the city of São Paulo contributes significantly to guaranteeing the access of the HP to health services and broadens the possibilities of early detection, treatment, follow-up, cure of chronic and infectious diseases, promoting humanized, comprehensive, longitudinal care and social reintegration.
